# The emergence of the stem cell niche

**DOI:** 10.1016/j.tcb.2022.07.003

**Published:** 2022-08-04

**Authors:** Michael R. Hicks, April D. Pyle

**Affiliations:** 1 Physiology and Biophysics, University of California, Irvine, Irvine, CA, USA; 2 Microbiology, Immunology, and Molecular Genetics, Eli and Edythe Broad Center of Regenerative Medicine and Stem Cell Research, University of California, Los Angeles, Los Angeles, CA, USA

## Abstract

Stem cell niches are composed of dynamic microenvironments that support stem cells over a lifetime. The emerging niche is distinct from the adult because its main role is to support the progenitors that build organ systems in development. Emerging niches mature through distinct stages to form the adult niche and enable proper stem cell support. As a model of emerging niches, this review highlights how differences in the skeletal muscle microenvironment influence emerging versus satellite cell (SC) niche formation in skeletal muscle, which is among the most regenerative tissue systems. We contrast how stem cell niches regulate intrinsic properties between progenitor and stem cells throughout development to adulthood. We describe new applications for generating emerging niches from human pluripotent stem cells (hPSCs) using developmental principles and highlight potential applications for regeneration and therapeutics.

## Introduction

Precise control over stem cell self-renewal and differentiation is essential for proper organogenesis and tissue homeostasis. Immediately juxtaposed to stem cells are specialized microenvironments, termed ‘niches’, containing multiple cell types guiding stem cell behavior. Just as adult stem cells are dynamically regulated by their niche, embryonic niches formed in development equivalently control cell fate decisions to mature from an early precursor or progenitor and eventually to an adult quiescent stem cell. Importantly, embryonic niches also change over these developmental contexts. This review seeks to identify how changes during development in both the stem cell and its niche affect behavior and characteristics of one another. We examine the idea of the emerging niche, defined as initial niches in development that help to capture or support fetal progenitor cells and direct their proliferative nature and eventual generation of quiescent adult stem cells. We specifically focus on niche emergence in skeletal muscle, but we highlight universal themes in emerging niche biology broadly across multiple stem cell systems and compare these with adult niches.

## Overview and importance of emerging stem cell niches in multiple systems

The initial niches formed in development are likely the most regenerative and pristine but may evolve over a lifespan or become altered in disease. Niches across tissue systems share common features, such as regulating progenitor to adult cell maturation, self-renewal, and activation states during regeneration.

A common theme for many niche systems is supporting waves of developing progenitors and stem cells. One of the best understood emerging niche systems is the hematopoietic stem cell (HSC) niche, which supports two HSC waves that give rise to all blood cells. The first primitive hematopoiesis wave produces transient hematopoietic cells to meet the immediate needs of the embryo, including primitive erythroid progenitors required for oxygenation. Definitive hematopoiesis then generates HSCs that give rise to all mature blood cells and have long-term repopulation potential.

Hematopoiesis occurs in discrete anatomical niches, including from the aorta-gonadmesonephros region, arteries, yolk sac, and placenta, that are age-matched for appropriate maturation and lineage specification [1,2]. Fetal HSCs seed the fetal liver, expand, and migrate to the adult niche in the bone marrow stroma, where they reside throughout adult life and become predominantly quiescent. As fetal HSCs mature, they begin to express receptors, including integrin-αM and GPI-80, that support migration from the liver and interaction with the stromal niche [3]. Interestingly, some definitive HSCs that originate from distinct fetal niches cease to persist into adulthood and instead are the cell of origin for tissue-resident innate-like lymphocytes [4]. In HSCs, the emerging niche is key for preventing alternate fate specification, such as to a cardiomyogenic fate [5]. The generation and maturation processes of HSCs during embryonic and neonatal development are primarily stimulated by inflammatory cytokines, which change over time, and there is still much debate on the role of intrinsic and extrinsic regulation of true HSC potential in the niche.

Interestingly, another common theme for many adult niche systems is reversion toward the developmental-like niche after an injury to support stem cell regeneration. The brain houses at least two well-studied neural stem cell (NSC) niches in the subgranular zone in the hippocampal dentate gyrus and the subventricular zone around the lateral ventricles [6,7]. A recent study performed single-cell profiling plus lineage tracing of mouse forebrain NSCs and showed that dormant NSCs reacquire developmental-like states when activated to generate adult-born neurons [8]. Notably, almost half of all transcriptomic differences were involved in regulating and/or sensing the niche environment, including cues from neurotransmitters, receptor ligands, and extracellular matrix (ECM) proteoglycans. Bidirectional switches between activated and dormant states during brain development or regeneration may be determined by the niche environment, including proper vascular formation, ECM, and supportive niche cells such as microglia that secrete neurogenic factors.

## The making of the stem cell niche in skeletal muscle

The adult skeletal muscle SC niche is a precise anatomical location between the plasma membrane of myofibers and a laminin-rich basal lamina. In contrast, emerging niches of skeletal muscle loosely consist of minimal basal lamina and immature myofibers that enable progenitor expansion of the rapidly growing muscle [9,10].

Skeletal muscle precursor/progenitor cells (SMPCs) in trunk and limb originate through segmentation of the paraxial mesoderm, leading to somitogenesis, which results in the formation of distinct anterior/posterior compartments important for specification of the dermomyotome and myotome structures that produce embryonic SMPCs. Key components of the dermomyotome and myotome niches, including signals from the emerging neural tube/ectoderm and mesenchyme, are reviewed elsewhere [10].

**Figure 1.**
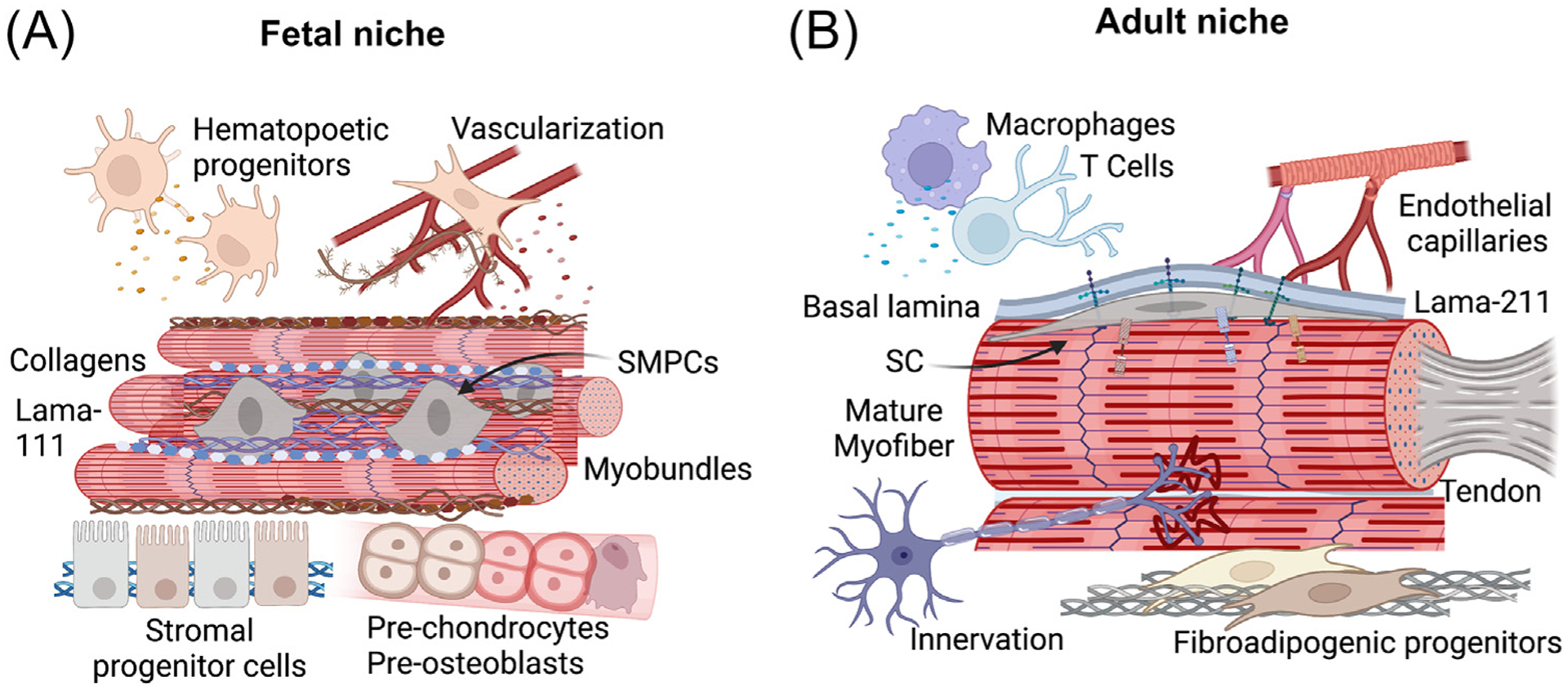
The emerging fetal and adult skeletal muscle niche. Shown is an example of differences between (A) fetal skeletal muscle precursor/progenitor cells (SMPCs) at the beginning of secondary myogenesis and (B) adult skeletal muscle satellite cells (SCs) within their niche and microenvironment [26]. Multiple SMPCs in the emerging niche illustrate increased number or set point. SMPCs adhere to clusters of small secondary myofibers termed ‘myobundles’, and SCs reside at the periphery of large adult myofibers with a laminin-rich basal lamina. In contrast, SMPCs produce high levels of extracellular matrix proteins and are in direct contact with multiple nonmyogenic cell types that are unique from the adult niche.

Once embryonic progenitor cells emerge from the myotome, one of the first steps in the formation of the niche is cell–cell adhesion between SMPCs and the sarcolemma of newly formed myofibers through interactions with M- and N-cadherins and CD82 [11,12]. This interaction occurs prior to the formation of the basal lamina (M.R. Hicks *et al*., unpublished). As multiple myofibers aggregate to form myobundles, a subset of progenitors become encompassed by fusing myofibers, noted by spectrin-positive cross-bridges [13] (Figure 1). Between human fetal weeks 11–13 (approximately embryonic day e14 in mouse), myofibers begin to express basal lamina such as laminin-211 that ensheathes the myobundles and associated progenitors to form the fetal niche [14,15].

Fetal SMPCs produce an order of magnitude greater level of ECM than adult SCs, which contributes to autonomous niche building [16]. The fetal niche includes fibronectin, collagens, nidogens, matrix metalloproteases, tenascin C, and others that provide scaffolding for fetal SMPCs and also serve as a reservoir for growth factors such as insulin-like growth factor 1 and transforming growth factor-β superfamily members to promote fetal muscle hypertrophy [19,20] and fibroblast growth factors (FGFs) essential to SMPC expansion [17–20]. The composition of fetal ECM differs from the adult niche, which includes embryonic laminin isoforms LAMA511 and LAMA111, which may facilitate formation of new myofibers [15,21]. ECM composition also influences niche stiffness, allowing loose association of the SMPCs to the myofibers through multiple binding sites, including fetal integrins such as integrin-α6 [22], and potentially regulates lineage commitment through YAP/TAZ signaling.

Over development, the skeletal muscle dynamically changes to support three waves of myogenesis in part by tendon connections that apply tensile forces [23] and motor neuron innervation leading to contraction [24] and structural maturation of the myofiber. The first primary skeletal muscles arise in the limb from e10.5 to e12 in the mouse [25] or weeks 6–9 in humans [26] to establish basic muscle patterning and are usually considered to be slow-twitch fibers marked by embryonic *MYH3* [27]. Primary myofibers support adjacent progenitors in the limb that express PAX3 and the transition to early PAX7 progenitors [28]. Motor neurons emerge from the ectoderm in weeks 6–9 in the human limb [29] and continue to mature and innervate in concert with the formation of secondary fetal myofibers. Secondary myofibers transition to fast-twitch fibers characterized by fetal myosin *MYH8* and fetal actin *ACTC1* that support the rapid expansion of fetal PAX7+ SMPCs and contribute to growing muscle. Secondary myogenesis is thought to occur around e12–e16 in mouse limbs and corresponds with innervation in humans by weeks 9–11 [30,31], but timing may vary depending on anatomical location. Secondary myofibers are initially polyinnervated and express fetal acetylcholine subunits, but this begins to decrease at fetal week 16 in humans [30]. Interestingly, fetal acetylcholine receptors are re-expressed during regeneration throughout adult muscle [32], similar to what is seen in other niche systems. Structural maturation of fetal myofibers corresponds with changes to their plasma membrane, including expression of potassium-gated ion channels and calcium channels such as Piezo1 that regulate sarcolemma phospholipids and begin to assemble the components needed to build the SC niche [33,34]. Adult myofibers of the limb are made up of multiple myosins, which include *MYH1* and *MYH2*, unless they are undergoing regeneration, in which case they re-express embryonic and fetal myosin isoforms, such as what is seen in emerging niches in development.

## Microenvironment of emerging niches

Emerging niches are also composed of several nonmyogenic cell types in the developing limb that are nearby or in contact with SMPCs (Figure 1). Prominent cell populations include preosteogenic SHOX2+ progenitors and prechondrogenic SOX9+ progenitors involved in bone or cartilage formation, respectively [26,35]. SHOX2-deficient limbs have aberrant neural and muscle formation during forelimb development [36]. The developing microenvironment also contains mesenchymal DUSP6+ progenitors that provide key morphogens needed for support and lineage specification of skeletal muscle [37,38]. DUSP6+ progenitors are multipotent but become more lineage restricted overtime, forming dermal TWIST2+ fibroblasts of the fascia, tenogenic TNMD+ cells that subdivide muscle masses [23], and fetal CD73+ stromal cells that differentially remodel the emerging niche. Fetal stromal cells coexpress platelet-derived growth factor receptor-α (PDGFRα), a marker for adult fibroadipogenic progenitors (FAPs), which are important support cells during adult SC regeneration [39,40]. However, fetal stromal cells differ from adult FAPs, including by increased hepatocyte growth factor and reduced interleukin-6 (IL-6) secretion, which regulate migration and expansion of SMPCs [43]. Also abundant in human fetal muscles are AIF1-expressing hematopoietic lineages that may give rise to tissue-resident macrophages [26]. Macrophage subtypes regulate SMPC activation, clearance of cellular debris, tissue immune surveillance, and the resolution of inflammation [41]. Model organisms have demonstrated that macrophages provide a transient muscle stem cell niche via nicotinamide phosphoribosyltransferase secretion during regeneration [42]. Table 1 provides a summary of emerging niche populations.

## Niche support of intrinsic differences between progenitor and stem cell states

Fetal progenitors and adult stem cells have key intrinsic differences that are tightly regulated by their niches. In skeletal muscle, both SMPCs and SCs express the quintessential transcription factor PAX7, but many other myogenic regulatory factors and signaling are markedly different. Adult SCs have tightly controlled transitions between quiescent and activated states that are PAX7+MYF5− or PAX7+MYF5+ [48]. When adult SCs express MYOD, they readily and robustly differentiate to myofibers. Unlike adult SCs, fetal PAX7+ SMPCs coexpress myogenic regulatory factors MYF5 and MYOD without differentiating into myofibers and are maintained in an activated state for extended periods of time, enabling expansion of the progenitor pool *in vivo* until the niche matures [49].

### Niche-regulated proliferation

One of the most striking differences in the fetal niche is the presence of amplifying progenitors in S/G_2_/M phases [50]. Progenitors are marked by high levels of EdU and Ki67 during limb development and at myotendinous junctions. Coinciding with proliferative differences, the fetal niche supports progenitors at densities 100-fold greater than the adult niche. Proliferative demands are reflected in SMPC metabolism, which is highly glycolytic and has mitochondrial respiratory capacity more similar to activated adult SCs after injury. The fetal niche tightly regulates low oxygen levels through hypoxia-inducible factor-1α and is used to drive proliferation and muscle mass [51]. Skeletal muscle growth is also particularly vulnerable in the fetus exposed to undernutrition from placental insufficiency. In contrast, adult SCs primarily metabolize through oxidative phosphorylation [26,52]. Adult stem cells maintain the quiescent G_0_ state for extended periods of time, although a limited number of SCs fuse with adult myofibers to maintain homeostasis [53]. Cell stress regulation induced by the niche is a key feature of quiescence that is required to prevent replication-associated mutations [54]. Key cellular responses to DNA damage pathway modulators, p53 and p16, have been shown to control quiescence across many adult stem cell systems not seen in fetal progenitors [55].

### Niche signaling

**Figure 2.**
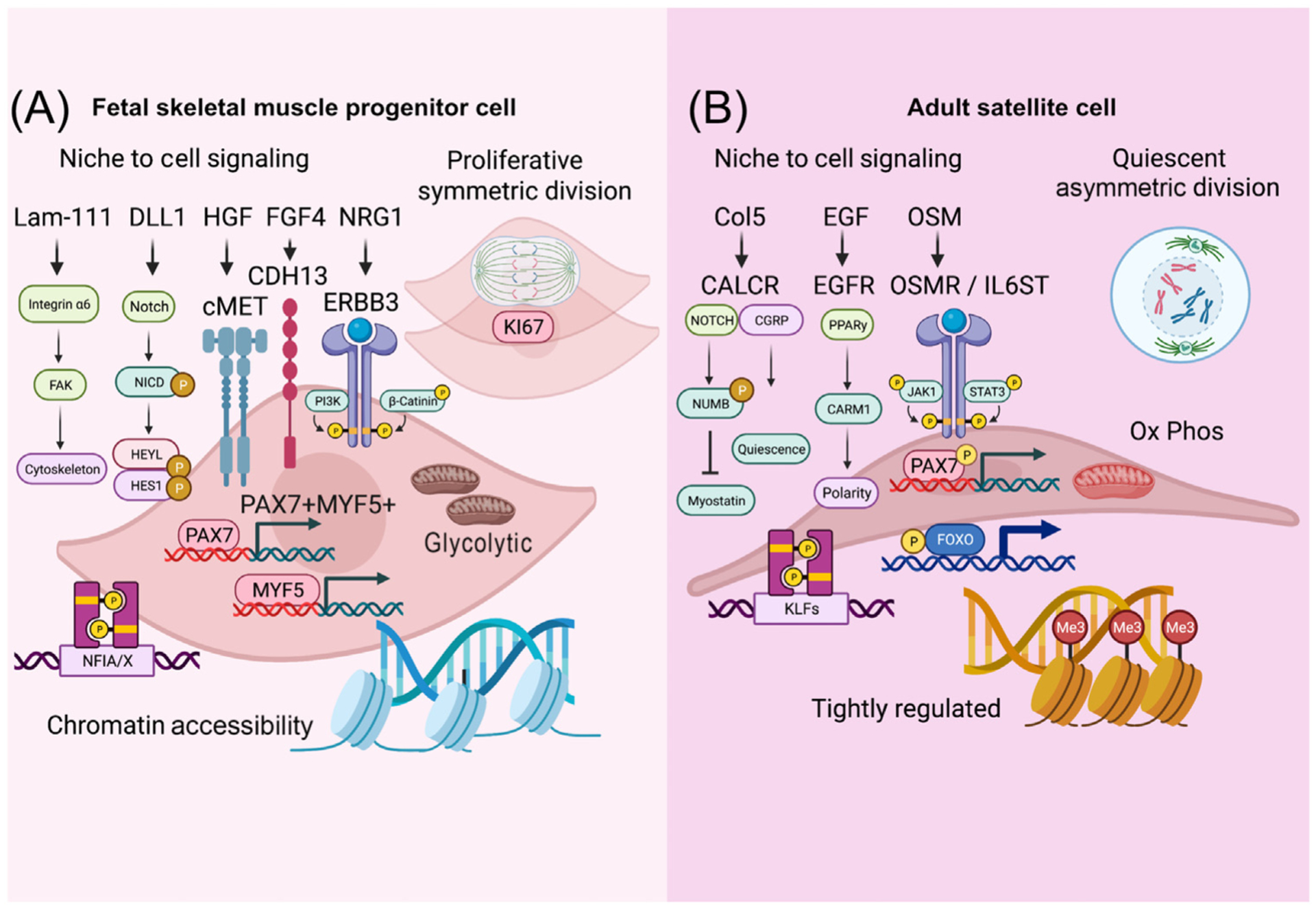
Intrinsic differences in skeletal muscle precursor/progenitor cells (SMPCs) and satellite cells (SCs). Cartoon depicts key differences in niche-derived signaling pathways identified between (A) SMPCs and (B) SCs. These ligands and receptors present on stem cells, or their niche cells, may influence cell behavior that includes proliferation, maturation, and quiescence.

Signaling pathways that regulate the interaction of the myofiber with progenitor or stem cells are key components helping to regulate the sequential colonization of the niche (Figure 2). Among the best-defined niche signals is the highly conserved Notch signaling system, which transduces short-range signals by interacting with transmembrane ligands such as Delta (DLL1) and Jagged-1 on neighboring cells [56]. While Notch signaling is a shared pathway between both SMPCs and SCs, Notch may interact with different coreceptors to induce niche colonization by SMPCs or maintain quiescence by SCs. Emerging SCs are driven into the interstitial space by a lack of Notch signals which is mediated through *Rpbj*. Mislocated SCs do not contribute to normal fiber growth in fetal development [14]. Another important signaling pathway are the integrins that interact with fetal and adult laminin isoforms that form the basal lamina of the niche. Integrin-α6 interacts with fetal laminin-111 and stimulates regeneration [22], whereas integrin-α7 and −β1 interact with adult laminin-211 but not embryonic isoforms [57] and are involved in several downstream signaling cascades, including focal adhesion kinases, cytoskeletal rearrangement, and RhoA [58], which regulates the quiescent state.

### Niche engulfment

As fetal myofibers undergo hypertrophy and fusion, the SMPCs adhered to the myofibers’ periphery become ‘engulfed’ by the niche. A multitude of receptors promote adhesion sites between fetal SMPCs and myofibers; these include M-cadherin, Mcam, Megf10, several α-integrins, and Vcam-1 [14,59,60]. Of these, Megf10 is an epidermal growth factor (EGF) repeat-containing transmembrane protein that is known for functioning as an engulfment receptor across tissues that can lead to actin polymerization [61] and, when overexpressed, can lead to cell engulfment of other cell types [62]. Megf10 is highly expressed in SMPCs and interacts with Notch and integrins, and Megf10 knockout mice have impaired muscle regeneration [63], which may in part be due to Megf10’s importance for SMPC engulfment into the niche.

### Niche-regulated cell polarity

Acquisition of cell polarity is an important parameter of stem cell maturation for many tissues, including the hair follicle, blood, nervous system, and skeletal muscle [64]. In HSCs, loss of the key polarity protein Lis1 prevents blood formation, resulting in embryonic lethality, and impairment of cell polarity in NSCs is linked to neurodevelopmental disorders such as Down syndrome, fragile X syndrome, autism spectrum disorder, and schizophrenia [65]. In the adult, when polarity goes awry, HSCs accelerate differentiation, which, in this context, prevents their ability to transform to myeloid leukemia by regulating inheritance of cell fate determinants [66].

Upon formation of the skeletal muscle niche, cells begin to establish an apical and a basal side, which enables polarity and asymmetric divisions of SCs both to form myoblasts and to self-renew. In contrast, fetal SMPCs are more prone to symmetrically divide [67]. Through an *in vitro* muscle stem cell niche screen, epidermal growth factor receptor (EGFR) and Aurora kinase pathways were identified as key determinants of SC polarity through peroxisome proliferator-activated receptor-γ and CARM1 [68]. In contrast, early fetal SMPCs do not express EGFR but highly express other family members of the EGFR pathway, such as ERBB3, which is shown to promote SMPC expansion and survival. ERBB3 is not expressed by adult SCs unless activated [69]. ERBB family members dimerize to affect downstream signaling, and the divergent expression between ERBB3 and EGFR and cosignaling pathways may represent a key transitory period between activation and quiescence states [70]. Indeed, as SCs are displaced from their niche, EGFR is among the first receptors to reduce expression [68].

### Niche receptors of SC quiescence

The calcitonin receptor (Calcr) is uniquely expressed by quiescent adult SCs and inhibits SCs from escaping the SC niche [71]. The Notch-COLV-Calcr signaling cascade maintains SCs in a quiescent state in a cell-autonomous fashion [72] that is thought to occur in mouse by 2 weeks postnatally [73]. Oncostatin M (OSM) receptor is a member of the IL-6 family and another important receptor of adult SCs. OSM is expressed by nascent myofibers in the niche 4–10 days after injury and is a potent molecule for reinduction of SC quiescence [74]. OSM stimulates the Stat3 pathway, which is among the most potent drivers of adult SC quiescence [75] and is weakly expressed or not expressed at all in fetal SMPCs.

### Stem cell niches regulate functional potential

In mouse, a single adult SC transplanted into a recipient muscle can self-renew to form many myofibers and re-establish the stem cell pool [76] and can be serially transplanted into multiple recipients with continued stemness [77]. This is the gold standard test for stem cells, as is shown in multiple stem cell systems, including the Lrg5+ stem cells in intestine [78] and epithelial progenitor cells in the skin [79], with strikingly similar outcomes. However, fetal SMPCs have limited self-renewal potential and poor survival upon transplantation *in vivo* and lack a strong ability to form adult myotubes *in vitro* [80]. It remains unknown whether these self-renewal defects are due to the fact that most transplantations are into matured adult microenvironments, such as during transplants of fetal SMPCs into adult muscle tissue, as it has not been tested *in utero*, where the developmental stage would more closely match the fetal niche. In other tissue systems, on the one hand, developmental timing has been shown to be important for cell competition following transplantation in which immature cells lose stemness when transplanted into more mature skin niches [81] or immature cells improve engraftment when transplanted into developing brain niches [82]. On the other hand, fetal SMPCs can be enriched at far greater numbers per gram of tissue than in the adult for therapeutic use but have limited true repopulation potential needed for use in cell-based therapies.

## Modeling emerging niche formation *in vitro*

Directed differentiation of hPSCs to skeletal muscle is among the few robust *in vitro* systems able to increase PAX7 expression by 1000-fold [83–85]. PAX7 cells not only are generated from hPSCs but also can be maintained in directed differentiation cultures for weeks to months [86]. Directed differentiation produces an *in vitro* niche that is not equivalent to the typical well-studied adult niche. Across many lineages, differentiation of hPSCs results in phenotypes more closely aligned with embryonic to fetal progenitor cells [26], and therefore, the niches that support hPSC muscle are more likely to be similar to an early developmental state. Just as with fetal development, an expected key component of emerging niches is the myofibers that are present in all directed differentiation strategies, and it is thought that secondary myogenesis in a dish can enhance maturation of Pax7 cells [87]. During directed differentiation, many nonmyogenic lineages concurrently arise that may serve supportive functions through secretion of growth factors, ECM, cell–cell contact, or mechanical cues to create an *in vitro* niche for PAX7 SMPC generation. Many supportive cells found in emerging fetal niches (Table 1) are also found during directed differentiation, including neural progenitor cells, neurons, and mesenchymal cells, and, depending on the protocol, can also contain epithelial cells, skeletal cells, and chondrocytes. Depending on the timing and directed differentiation strategy, the supportive cells that emerge may differ [26]. The role of these emerging populations has yet to be tested, but these populations have the potential to inform how to better generate or support PAX7 cells during human development.

A cutting-edge area of emerging *in vitro* niches may include engineering 3D human skeletal muscle systems to provide a more long-term environment for support of PAX7 cells. *In vitro* myobundles have been developed that resemble the fetal myobundle niche and that exhibit calcium transients and nearby PAX7 SMPCs [88]. Over a 4-week culture period, 3D induced skeletal muscle bundles undergo progressive myotube hypertrophy and functional enhancement and attain more advanced levels of myogenic differentiation than do age-matched 2D monolayers. In another format, induced myogenic progenitors (iMPCs) can undergo direct reprogramming and conversion to the myogenic program to generate spontaneously contractile myotubes with abutting Pax7+ cells near most fibers, suggesting the formation of *in vitro* emerging niches in an iMPC system [89].

Despite advances in generating PAX7 cells in 2D and 3D systems, there are currently still no protocols that can fully support or mature the equivalent of an adult muscle SC from hPSCs *in vitro*. Avenues to induce maturation could include addition of vascular cells, including endothelial cells, pericytes, or integration with motor neurons, to fully support generation of more mature adult SC-like cells *in vitro*. Recent work has shown that both muscle and neuronal cells can be generated in a new organoid system called the ‘neuromuscular organoid’ [90], but whether this organoid model better supports developing or more mature adult PAX7 cells is not known. Vascular support would also improve survival of larger constructs *in vitro* and *in vivo*, which require vascularization to prevent hypoxia-induced cell death. Towards this end, it has been shown that generation of 3D muscle models alone, with injury [114] or containing multiple supportive cells in addition to skeletal muscle, including vascular endothelial cells, pericytes, and motor neurons, can be generated, but the potential of PAX7 cells grown in 3D to engraft into the SC niche *in vivo* better than 2D conditions is still underway [91].

In addition, the *in vivo* environment may provide a better system for generating more mature and potentially quiescent adult SCs [92,93]. However, even in these conditions, the maturation does not occur without serial transplantation *in vivo* and/or after injury. Recapitulating the *in vivo* niche in a dish will be needed to overcome these hurdles and generate an SC equivalent without the need to mature these cells in animal models.

## Generating new niches for therapeutics

### Regulating the stem cell niche set point

Can we use the information gained from emerging niches to improve niche formation for use in regenerative medicine applications? Within any given organ, the niche tightly regulates the number of stem cells within it, termed the ‘niche set point’. The niche set point varies widely across organs; for example, the intestinal crypt, dental pulp, skin, and hair follicle bulge contain densely packed stem cells, whereas the liver and skeletal muscle niche contain more sparse stem cells [94]. Understanding the cues that regulate the stem cell niche set points could be harnessed to potentially increase stem cell numbers in the niche and the robustness of the regenerative response [95].

Emerging niches can be generated from hPSC SMPCs that recapitulate the early stages seen in muscle regeneration *in vivo* (M.R. Hicks et al., unpublished). During regeneration in the adult, new myofibers are formed, and PAX7 cells associate with regenerating myofibers [96]. Both in development and in regeneration, there are more SMPCs per unit of myofiber than at homeostasis, but whether we can regulate stem cell densities or set points is less well understood. This work suggests that a better understanding of the interaction of the SMPCs with the niche, perhaps in the context of emerging niche formation, similar to what is seen in regeneration, will provide clues as to how SMPCs can be remodeled to generate more functional SCs.

It is interesting that as niches in development support more progenitor cells than the adult, it could be possible to rejuvenate the aging or diseased niches by recapitulating the niche set points, such as those that are created in development. Recently, overexpression of the Yamanaka factors (Oct3/4, Sox2, Klf4, and c-Myc) in myofibers caused changes to the myofibers, which included upregulation of cytoskeletal reorganization pathways and induced the activation of SCs, which could be similar to the SMPC state and in turn accelerated muscle regeneration in young mice [97]. Regulation of the niche has also been accomplished using receptor–ligand combinations, such as using myofiber-derived Wnt4, which was shown to regulate the stem cell niche set point [98], and knockout of signaling pathways such as with Stat3 in the SCs that makes them nonresponsive to myofiber-induced signals such as with OSM [99]. Manipulating these or similar pathways in the emerging niches could help shift SMPCs toward an adult SC-like state.

### Modulating niche ECM/signaling

It has been shown that injecting niche factors that target the receptors and ECM can modulate regeneration. For example, monoclonal antibody augmentation of β1-integrin activity can restore Fgf2 sensitivity and improve regeneration after experimentally induced muscle injury [100]. To improve niche formation, it has been shown that treatment with fetal laminin-111 enhances symmetric cell division and improves regeneration in the adult, and this may harness aspects of a fetal-like regenerative state [15]. A powerful tool to enhance regeneration may be to develop artificial niches to support SCs *in vitro* [101]. Others have also targeted the supportive cells in the microenvironment to regulate the regeneration through Notch signaling [102], and depletion of niche cells reduces regeneration [103].

## Aging and diseased niches

It is important to note that the niche is not static but changes over the lifespan to regulate stem cell function in aging and disease contexts [104]. For example, in the aging intestinal niche, loss of barrier function in the epithelium alters stem cell fate [105], and shifts in the number of Paneth cells in the niche reduce regenerative potential. In neurogenic niches, high vascular density and disruption of vascular remodeling in early development may play a long-term role in regulating neural organization, and vascular cell loss in the brain may be a contributor to neurodevelopmental disorders such as autism, schizophrenia, and epilepsy [106].

Some of the worst muscle diseases occur from genetic defects in key components of the SC niche. Mutations in laminin-2 or collagen-6 genes result in congenital myopathies and Emery-Dreifuss disease, respectively, and lead to loss of Pax7 cells and muscle wasting [107]. In one of the most devastating muscle-wasting diseases, Duchenne muscular dystrophy (DMD), SCs deficient in dystrophin lose polarity and are unable to appropriately form a niche, leading to a dysfunctional progenitor state, failed regeneration, and/or disease exacerbation [108]. Recent work has shown that SCs survey their niche via cell protrusions [109], which, when impaired, such as in Piezo1 knockout or DMD, primes SCs to activate from quiescence [33].

A key unresolved question is whether defects in emerging niche formation lead to incomplete maturation of the stem cell or a propensity for birth defects or developmental delays. Convincing evidence has demonstrated that some pediatric leukemias can originate before birth and that these cancers retain fetal niche properties postnatally [110,111]. Understanding the ontology of fetal hematopoiesis is of particular interest in understanding the pathogenesis of childhood blood disorders [112]. Similarly, understanding how fetal skeletal muscle growth adapts to nutrient availability is important for determining deficits in muscle growth in adulthood. Because skeletal myofiber number is set at the time of birth, low birth weight infants may have lower muscle mass in adulthood, resulting in increased risk for metabolic syndrome and type 2 diabetes. Thus, suppressed development of muscle by inefficient niche formation or control could be a major contributor to increased risk of sarcopenia, obesity, or diabetes later in life [113].

## Concluding remarks

Stem cell niches range in biological diversity across development to adulthood. The niches not only promote regeneration but also regulate the maturation and functional potential of the progenitors or stem cells they support. Niches should be considered as a therapeutic target to readily influence both intrinsic and extrinsic activity of progenitors and stem cells in development and disease. hPSCs offer a powerful system to generate robust regenerative cells, and this could have implications for use across many different cell-based therapeutic applications, especially if they can be directed to generate fully functional adult SCs. Evaluation of the differences in emerging compared with adult niches using new tools, including spatial sequencing, multiomics, and niche-specific computational platforms, will accelerate our understanding of how progenitor and stem cells are controlled differently in each niche state. Harnessing the power of emerging niches could shed light on new approaches to enhance regeneration and support human health (see Outstanding questions).

## Figures and Tables

**Table 1.** Supportive cell types in the emerging niche.^a^

Cell populations in the emerging niche	Markers	Potential roles in emerging niche formation
Fetal myofibers	MYH8, ACTC1, spectrin	Adhesion to fetal SMPCs, building of ECM needed to support SMPCs
Prechondrocyte	SOX5, SOX6, SOX9	Specification and support of muscle development, BMP production
Preosteoblast	SHOX2	Specification and support of muscle [36]
Limb mesenchyme	DUSP6	Wnt regulation and limb formation
Tendon precursors	SOX9, TNMD, SCX	Skeletal muscle patterning and tensile forces inducing structural maturation [23,37]
Dermal fibroblasts	TWIST2, KRT19	Chromatin remodeling and maturation [43], formation of the fascia
Stromal cells	CD73, PDGFRα	Regulation of immune system and SMPC activation through cytokine section and adipocyte versus fibrotic differentiation [44]
Neural crest	SOX10, Wnt1	Prevention of premature differentiation of fetal SMPCs [45]
Schwann cells	CDH19	Neuromuscular junction formation and maintenance [46]
Vascular and endothelial cells	ESAM, AP	Metabolite and growth factor support to the skeletal muscle [47]
Macrophages	SRGN, AIF1	Regulates muscle regeneration, stromal cell activation and maturation

a Abbreviations: BMP, bone morphogenetic protein; ECM, extracellular matrix; SMPC, skeletal muscle precursor/progenitor cell.

## Highlights

Niches coevolve with the maturation of progenitor and stem cells from development through to adulthood.

Fetal progenitors in emerging niches are highly expansive, whereas adult niches regulate stem cell quiescence.

Fetal progenitors are less able to occupy adult niches during transplantation.

Niche formation is stepwise, involving cell–cell contact, followed by cell– extracellular matrix contact, to affect cell polarity and maturation.

Emerging niches use similar pathways and mechanisms during regeneration.

Understanding niche formation may improve the quality and quantity of stem cells within niches.

Supportive cell types in emerging niches are different from homeostatic adult niches.

Human pluripotent stem cell lineages can be used to model components of niche development *in vitro* and *in vivo*.

## Outstanding questions

What dictates progenitor differentiation versus niche formation?

How does adherence to the emerging niche make the transition to adult SC niches over time?

How do SMPCs transition into a quiescent SC?

How many transitional states are there between fetal progenitor and adult stem cells?

What regulates stem cell set point and number of niches across tissues?

What are the roles of supportive cells in the emerging progenitor niche compared with the adult niche?

What are similarities and differences between development and regeneration?

Can new niches be rejuvenated in settings of aging or disease?

Would the formation of new niches improve the rate or magnitude of regeneration?

Is the myofiber plasma membrane specialized at the myofiber SC contact point, and how does this regulate SC set points?

Can maturation of hPSC SMPCs be promoted by modulating the niche?

What are the cues that are driving maturation *in vivo*?

Are the processes that regulate the formation of the first niches from hPSCs the same as *in vivo* emerging niches?
